# The Application of Bioreactors for Cartilage Tissue Engineering: Advances, Limitations, and Future Perspectives

**DOI:** 10.1155/2021/6621806

**Published:** 2021-01-21

**Authors:** Liwei Fu, Pinxue Li, Hao Li, Cangjian Gao, Zhen Yang, Tianyuan Zhao, Wei Chen, Zhiyao Liao, Yu Peng, Fuyang Cao, Xiang Sui, Shuyun Liu, Quanyi Guo

**Affiliations:** ^1^Institute of Orthopedics, Chinese PLA General Hospital, Beijing Key Laboratory of Regenerative Medicine in Orthopedics, Key Laboratory of Musculoskeletal Trauma & War Injuries PLA, No. 28 Fuxing Road, Haidian District, Beijing 100853, China; ^2^School of Medicine, Nankai University, Tianjin 300071, China

## Abstract

Tissue engineering (TE) has brought new hope for articular cartilage regeneration, as TE can provide structural and functional substitutes for native tissues. The basic elements of TE involve scaffolds, seeded cells, and biochemical and biomechanical stimuli. However, there are some limitations of TE; what most important is that static cell culture on scaffolds cannot simulate the physiological environment required for the development of natural cartilage. Recently, bioreactors have been used to simulate the physical and mechanical environment during the development of articular cartilage. This review aims to provide an overview of the concepts, categories, and applications of bioreactors for cartilage TE with emphasis on the design of various bioreactor systems.

## 1. Introduction

The regeneration of articular cartilage (AC) is one of the challenges in regenerative medicine due to its poor regenerative capacity [1, 2]. Natural AC is a complex hierarchical structure that is avascular with four layers: the surface zone, middle zone, deep zone, and calcified zone [3]. These zones have different biochemical compositions, chondrocyte phenotypes, and physiological characteristics tied directly to the effects of mechanical loading and the physiological environment [4, 5]. The current clinical treatment strategies mainly include arthroscopic debridement, microfracture, and autogenous osteochondral transplantation, which can promote tissue recovery to some extent, but the quality and long-term repair effect of regenerated tissue are not satisfactory [1, 6, 7].

Tissue engineering has brought new hopes for AC regeneration, as it can provide structural and functional substitutes for native tissues [8, 9]. The basic elements of tissue engineering involve scaffolds, seed cells, and biochemical and biomechanical stimuli [10, 11]. According to the presence of seeded cells, scaffolds can be divided into cellular scaffolds and cell-free scaffolds [12]. There have been many studies on the three-dimensional porous biodegradable structure of scaffolds for chondrocyte or mesenchymal stem cell (MSC) seeding [13, 14]. However, there are many limitations of cell-seeded scaffolds, one of the most important being that static cell culture on scaffolds cannot simulate the physiological environment required for the development of natural cartilage [15]. For example, in addition to the uneven delivery of oxygen and nutrients, the associated lack of mechanical stimulation causes decreased proliferation and differentiation and results in nonuniform cell distribution. For optimal AC repair, tissue-engineered cell-seeded scaffolds should be structurally and functionally similar to normal AC, and the surrounding physiological environment that can influence chondrogenesis should be modeled [16, 17]. The environment includes hydrostatic pressure; fluid shear force; microgravity; sound waves; magnetic field; and biochemical conditions, such as the pH, CO2, and pO2 levels; temperature; and growth factors. Therefore, traditional cell culture techniques cannot meet the requirements of AC tissue engineering, and complex bioreactors have recently been used to simulate the physical and mechanical environment that influences chondrogenesis [3, 18, 19]. Bioreactors are powerful dynamic culture model systems that can apply a combination of chemical, mechanical, electrical, and/or magnetic stimulation to study the basic mechanisms of cell function under physiologically related conditions within seeded cells and to facilitate the correct development of tissue [20, 21].

The aim of this review is to summarize the bioreactors that have been applied in tissue engineering articular cartilage (TEAC). In detail, we introduce the common categories and important design principles of bioreactors. In addition, we discuss the combinations between bioreactors and some techniques, such as 3D printing and microcarriers. Subsequently, we consider the challenges and future prospects for bioreactors in TEAC.

## 2. Common Types of Bioreactors

An ideal bioreactor should have several necessary functions, including enhancing the expansion of seeded cells, promoting the exchange of nutrients and oxygen, and providing appropriate physicochemical stimuli [16, 19]. Currently, the most common types of bioreactors are spinner flasks, rotating wall vessels, and perfusion systems [19]. Moreover, with the development of TEAC, some novel bioreactors that provide noncontact biomechanical loads have been designed, such as magnetic bioreactors and ultrasonic bioreactors. The application of these bioreactors (as shown in Table 1) aims to simulate the complex physiological and biomechanical environments in AC [3].

### 2.1. Spinner Flasks (SFs)

**Figure 1:**
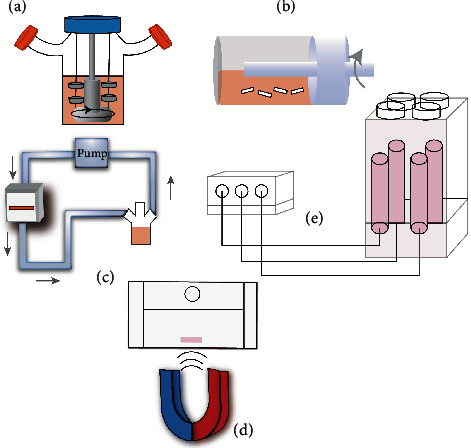
Common bioreactors for tissue engineering. (a) Spinner flask. (b) Rotating wall vessel. (c) Direct perfusion. (d) Magnetic bioreactor. (e) Ultrasonic bioreactors.

An SF is a cylindrical culture system that includes two arms used to remove the stumps and a bottom stirring system to mix the culture medium (Figure 1(a)) [19, 22]. Scaffolds are mostly hung from the top of the flask with a needle or thread. SF bioreactors can create fluid convection and hydrodynamics that enhance the efficiency of nutrient delivery and seeding of cells in the scaffold. Moreover, numerous studies have shown that SFs can be used in AC tissue engineering for AC tissue formation and cell function [19, 23]. For example, Yoon et al. used SFs to culture cartilage formation of human ADSCs (hADSCs), which were induced toward spheroid formation and chondrogenic differentiation on a large scale. The results showed that chondrogenic differentiation in vitro and subsequent chondrogenic formation in vivo of hADSCs in the spherical culture system was enhanced compared with those in monolayer culture. Therefore, the SF was found to promote the chondrogenic differentiation of hADSCs in the experiment, and its function in the large-scale culture of cells in AC tissue engineering was also verified [24]. In another study, Xu et al. applied SFs in association with chondrocyte seeding on alginate gel beads. It was suggested that 3D dynamic culture can provide a suitable system supporting both differentiation and redifferentiation of chondrocytes [23].

Although SFs present some advantages in the delivery of medium when compared with static culture, the degree of delivery is far from sufficient, and the shear force produced by the fluid can also adversely affect the cells.

### 2.2. Rotating Wall Vessels (RWVs)

RWVs consist of a pair of concentric cylinders: rotating outer cylinders to hold scaffolds or cells and inner cylinders that are static and used to exchange gases (Figure 1(b)) [19, 22]. Compared with spinner flasks, RWVs create a microgravity environment and provide controlled oxygen transport, as well as low shear forces and turbulence. For instance, Zhu et al. developed a cell-hydrogel cartilage construct in a dynamic RWV. The results suggested that the mass transfer efficiency of RWVs was greater than that of static culture conditions in achieving final equilibrium [25]. In another study, Nordberg et al. investigated how simulated microgravity and cyclic hydrostatic pressure modulate chondrocyte homeostasis, specifically focusing on the effects of these mechanical stimuli on LRP4/5/6 expression, which has been shown to be essential for maintaining the balance between bone formation and resorption. They simulated rat chondrosarcoma cell (RCS) pellets under cyclic hydrostatic pressure (1 Hz, 7.5 MPa, 4 h/day) or simulated microgravity in an RWV bioreactor. The experimental results showed that the growth of RCS particles was regulated by mechanical stimulation and that the expression of LRP4/5/6 in chondrocytes was affected under cyclic hydrostatic pressure or microgravity [26]. However, when the density and size of cells reach a certain level, collisions between the cells and the inside and outside walls of the RWV are inevitable. The collisions potentially induce large shear stresses that may be destructive to the cells in tissue engineering.

### 2.3. Perfusion Bioreactors

As described above, SFs and RWVs are not able to effectively infuse the medium into the scaffold. To overcome the limitations described, flow perfusion has been used for the regeneration of AC. Perfusion bioreactors use a pumping system to feed medium directly to scaffolds. Most of them consist of a pump, a culture media reservoir, a tubing circuit, and a perfusion cartridge that holds the scaffolds (Figure 1(c)) [20, 21]. The perfusion cartridge is one of the most important components that needs to be customized according to the properties of the scaffolds so that the medium can be injected directly into the scaffold instead of flowing around it. Moreover, the scaffold also needs to have highly connected pores. Recently, many perfusion bioreactors have been developed and applied in tissue-engineered cartilage. For example, Theodoridis et al. prepared a porous 3D-printed PCL scaffold and seeded it with adipose MSCs. Then, the scaffolds were cultured in a static culture and perfusion bioreactor, and the results showed that dynamic culture favored chondrogenic differentiation and achieved better penetration of cells within the scaffold than static culture [27]. In addition, Pigeot et al. used a perfusion bioreactor to culture human mesenchymal stem cells (hMSCs) seeded on collagen sponges and showed that a large amount of apoptosis-devitalized hypertrophic cartilage extracellular matrix (ECM) could be engineered using bioreactor systems [28].

### 2.4. Magnetic Bioreactors

The three bioreactors mentioned above mainly use mechanical stimuli on a macroscopic scale, such as hydrostatic pressure, shear force, tensile force, and compression force, which are directly applied to tissues or scaffolds to guide the behavior of cells. The limitations are the smaller range of stimuli and disadvantages of open culture conditions. Therefore, microscale mechanobiological techniques such as magnetic forces have been studied. Recent studies have found that magnetic fields affect cells, tissues, and entire organisms, including the formation of the extracellular matrix of hyaline cartilage, proliferation of bovine chondrocytes, and the synthesis of proteoglycan [29, 30]. Magnetic field bioreactors are advantageous, because they can meet the requirement of sterile culture conditions by contactless culture [31, 32]. Most of them consist of one or a group of permanent magnets that influence the behavior of cells through static or dynamic magnetic field strengths (Figure 1(d)). In addition, advanced magnetic bioreactors were designed according to the physical principles describing translational and rotational motion of magnetic particles, ferrofluids, and materials in high-gradient magnetic fields [33]. For example, Brady et al. constructed a novel high-throughput magnetomechanical stimulation bioreactor. Validation tests showed that the bioreactor provided a unique platform for researchers to study the combined effects of electrical phenomena and mechanical stimulation [32]. In another study, however, Dikina et al. investigated a variable magnetic field bioreactor composed of permanent magnets that were used for the culture of scaffold-free, high-density hMSC sheets, and the results showed that the bioreactor did not enhance chondrogenesis in the cell-only sheets. Therefore, more studies should be performed to validate the enhanced ability of chondrogenesis in response to magnetic fields [31].

### 2.5. Ultrasonic Bioreactors

Ultrasonic stimulation has also been shown to influence cell growth in some cases. In particular, low-intensity continuous ultrasound (US) has been shown to modulate the expression of chondrocyte-specific genes [34, 35]. The traditional ultrasonic bioreactor is realized by adding an ultrasonic processing system. With the development of technology, various ultrasonic bioreactors have been designed. Subramanian et al. designed a representative US-assisted bioreactor (Figure 1(e)). They placed a plate holder which retains the six-well plates with scaffolds above the transducer array. A custom splitter which allows manipulation of the ultrasound (US) signal makes all wells have identical pressure profiles. This apparatus is then used to stimulate chondrocyte maintenance. The tests showed that the bioreactor had positive effects on cell proliferation, viability, and selection of chondrocyte markers for gene expression [36]. In another study, Thakurta et al. placed chondrocyte-seeded scaffolds in the same US-assisted bioreactor, which supplied resonance frequencies (~5.0 MHz). The results showed that the COL2A1/COL1A1 ratios, ACAN mRNA, and the expression of Sox-9 were elevated compared to controls [34].

hADSCs: human adipose-derived stem cells; RCS: rat chondrosarcoma cells; hBM-MSCs: human bone marrow-derived mesenchymal stromal cells; PCL: polycaprolactone.

## 3. Considerations for Seeded Cells in Bioreactor Design

As a dynamic and static combination of three-dimensional cell culture device, bioreactor can simulate the microenvironment of cartilage in vivo and provide suitable external conditions for the construction of tissue-engineered cartilage by regulating the biophysical factors of seed cells. In this section, the latest development of bioreactors under different biophysical factors and the detailed design elements are described from the aspects of oxygen concentration, mechanical stimulation, and noncontact physical stimulation (as shown in Table 2), which are expected to provide some reference for the design of bioreactor.

### 3.1. Oxygen

Cartilage is already exposed to relatively low levels of oxygen during the initial stages of embryonic development. Therefore, studies have been conducted on the role of oxygen tension in cartilage formation. Numerous studies have shown that the biochemical components of cartilage in vitro are more similar to natural tissue when cultured under hypoxic conditions [37, 38]. Therefore, it is necessary to determine the standardized oxygen concentration in the bioreactor design. Increasing evidence has demonstrated that low oxygen tension promotes cell proliferation [39], enhances chondrogenic differentiation capacity [40], and increases migration ability [41] but inhibits osteogenic differentiation [42]. However, not all chondrocytes benefit from hypoxia. Kean et al. showed that hypoxia, while beneficial for articular-derived chondrogenesis, is detrimental for auricular-derived chondrogenesis under the same conditions [43].

Gruenloh et al. found that hypoxic (3.0% oxygen) treatment promoted in vitro migration of the human embryonic stem cell (hESC) line H9 (H9-MSCs), as demonstrated by significantly stronger cell mobility efficiency than that under normoxic conditions (5.0% CO2/ambient O2) [44]. Boyette et al. found that hypoxic treatment (5% oxygen) of human SDSCs prevented senescence induction, evidenced by 2.70-fold-lower (*p* < 0.01) expression levels for P16 in low oxygen tension-tissue engineering cartilage (LOT-TEC). They also found that hypoxia promoted cellular proliferation and enhanced chondrogenic differentiation [40]. Meyer et al. showed through direct comparison that low oxygen conditions are a more potent prochondrogenic stimulus than dynamic compression [45]. In contrast, there have been reports of negative effects of severe hypoxia on proliferation and differentiation of bone marrow mesenchymal stem cells (BM-MSCs). This may be because the pO2 is so low that BM-MSCs have to slow down their differentiation through molecular mechanisms [46]. This is why we need to choose more accurate oxygen tension conditions in the bioreactor to simulate the intra-articular environment.

There is no doubt that oxygen concentration is a very important culture parameter. By controlling the oxygen production concentration in a bioreactor, the fate of cells can be controlled in the desired direction.

### 3.2. Mechanical Stimulation

**Figure 2:**
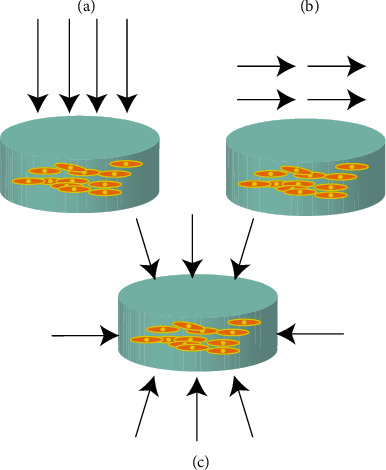
The arrows indicate the direction of the mechanical load acting on TEAC. (a) Compression. (b) Shear. (c) HP.

Mechanical stimulation plays an important role in the gene expression of seed cells in bioreactors. Seeded cells in scaffolds are not able to achieve the same biomechanical and histological properties as natural AC tissue without mechanical stimulation. In this section, mechanical stimulation is classified into three types: hydrostatic pressure (HP), shear, and compressive forces (CFs) (Figure 2) according to the different stress ranges and directions of TEAC to mechanical load. Moreover, we summarize the mechanical transduction mechanism and the design of bioreactors applied by different mechanical stimuli in AC tissue engineering. Finally, some of the latest research progress is discussed.

#### 3.2.1. Hydrostatic Pressure

Synovial fluid in the articular cavities of the knee joint and hip joint not only provides the necessary nutrients but also lubricates the joint surface. In the daily activities of humans, the surface tissues and cells of AC are subjected to fluid shear and HP in the liquid environment. Therefore, body fluids are an indispensable biomechanical condition in the construction of TEAC. Vunjak-Novakovic et al. suggested that the hydrodynamic conditions in bioreactors can enhance the secretion of GAG and collagen in the ECM of chondrocytes and regulate the morphology, mechanical, and electromechanical properties of tissue-engineered cartilage [47].

HP is the most common mechanical stimulation of the fluid environment. Endogenous AC bears intermittent HP, which is produced during human exercise. Tissues and cells are subjected to compression forces of uniform range and multiple directions under HP. The range of HP in AC is approximately 5–6 MPa upon walking and can reach 18 MPa upon running and jumping [48]. In addition, the HP frequency accompanies the human walking rhythm, which is usually no more than 1 Hz [49]. Therefore, in the design of HP-related bioreactors, the range and frequency of HP should be controlled within the above normal physiological range as much as possible. Because HP essentially does not shear or deform tissue, the damage of HP to the ECM is minimal for all mechanical stimuli in the joint. In recent years, the study of HP has been a hot topic in the field of tissue engineering cartilage, where it is considered the most important mechanical load [50]. HP is increasingly being recognized by researchers as an effective method for the cultivation and stimulation of chondrocytes in monolayer, three-dimensional (3-D) engineering structures, as well as in explants [51].

For the design of HP bioreactors, there are two main methods of applying HPs to cells: explants and constructs. In explants, HP transmits liquid pressure through the gas phase of the compressed medium. Since the oxygen tension in the pressurized medium changes as the gas phase is compressed, there are some limitations in the construction scheme of HP in the explants. The other method for constructs applies high pressure by compressing the liquid phase only, which prevents the solubility of oxygen in the medium from changing, as the pressure does not change during compression. This method cannot be used to examine the effect of HP on TEAC at different oxygen levels. The method for constructs usually involves connecting a fluid-filled chamber to a piston on a controllable hydraulic press through a hose [51]. These two different types of HP applications have their own advantages and disadvantages, and researchers can choose one or the other based on whether the HP experiment needs to be combined with changes in oxygen partial pressure.

Through meta-analysis, Hodder et al. concluded that static HP with a reaction time of more than 2 weeks in the physiological range of medium and high cartilage (≤5-10 MPa) is an ideal condition for tissue-engineered cartilage to produce large amounts of proteoglycans [52]. In another study, Cheng et al. found that HP stimulation of 0.12 MPa for 1 h/day for 4 days could significantly improve the function of BMSCs secreting cartilage matrix in rabbit BMSC/PRF (platelet-rich fibrin) construct during the process of chondrocyte differentiation [53]. Similarly, cyclic HP (0.5 Hz, 5 MPa, 4 h/day for 1 week) was found to significantly improve the chondrogenic differentiation ability of hBMSCs in demineralized bone matrix scaffolds [54]. HP is a mechanical load that can be easily applied to the large-scale construction of engineered cartilage due to its uniform effect on cartilage tissue. However, mechanical stimulation of HP is insufficiently multiform to form normal physiological layered cartilage through this loading type alone. Existing HP bioreactors have preliminarily incorporated techniques regarding different HP conditions on cartilage formation and cartilage, and researchers may focus on the regulation of cellular behavior in HP research in the future. Further specific review of the role of hydrostatic forces in regulating cell behavior was provided by Liu et al. [55].

#### 3.2.2. Shear

Shear is a load parallel to the AC surface generated by a liquid or solid medium during normal physiological activity. When cartilage tissue is compressed, there is a potential fluid shear force in and around the cell membrane during the discharge of large amounts of water. Then, with the compression force removed, the discharged water returns to its original position due to the osmotic pressure change and produces corresponding fluid shear stresses again [56, 57]. In addition, the proximal and distal sliding of the joint, such as the femoral condyle and tibial plateau in the knee joint, produces direct shear forces. Generally, it is suggested that continuous shear force is harmful to AC in vivo. The cartilage surface gradually wears away and degrades, and the possibility of inducing OA increases during the continuous application of shear force. Unlike the above, in the field of tissue engineering, shear stimulation has been used by researchers to improve TEAC structural properties in vitro. Past studies have shown that shear forces can increase cartilage matrix composition [58, 59], promote tensile properties [58], alter the expression of integrin [60], and reduce the friction coefficient under appropriate magnitude and frequency stimulation [61]. Oscillating shear is a common loading method in fluid and direct shear bioreactors, which can make TEAC have similar structure and mechanical properties as natural cartilage [62, 63]. By searching and summarizing the research articles related to cartilage tissue engineering and computer modeling, Pearce et al. concluded that computational modeling can help better characterize the liquid shear stress of the bioreactor, improve the scaffold structure, and control the mechanical properties of tissue-engineered cartilage through further modeling the growth, development, and movement of cells [64].

Nazempour et al. designed a bioreactor system that combined shear stress and oscillating hydrostatic pressure (OHP). The results showed that the secretion of GAG and collagen by chondrocytes was significantly higher than that of static culture, and the effect was better when both mechanical stimuli were applied simultaneously. In addition, low expression levels of -1 integrin and type-X collagen genes were found. The shear force and OHP may have protective effects on the cartilage due to the increase in the elastic modulus measured by atomic force microscopy under the combined load [59]. The shear load is applied only in the direction parallel to the chondral surface, corresponding to the formation of a horizontal AC surface; thus, there is a significant deficiency in the construction of TEAC using shear force alone as a mechanical stimulus aid. To produce high-quality engineered cartilage, additional mechanical loads must be superimposed. In recent years, most of the improved bioreactors have been designed for multiaxial loading, providing new insights into the responses of chondrocytes and chondrogenic stem cells to specific types of loading (especially shear).

For the design of shear bioreactors, according to the properties of mediators required for the transmission of shear load at the cartilage tissue level, shear has been divided into fluid-induced shear and tissue shear. Fluid shear bioreactors simulate the load on cells and tissues during the flow of synovial fluid and intercellular fluid in the body. Agitation systems in liquid environments, such as rotating flasks and direct perfusion systems, apply high shear forces, while RWVs apply low shear forces [65]. Bonnevie et al. found that high shear forces were closely associated with mitochondrial dysfunction, apoptosis, and cell death [66]. The mechanical stress in a low-shear bioreactor is usually less than 0.1 Pa, which was proven to increase extracellular matrix content and cartilage formation without damaging cells [58, 67]. Shear force between tissues usually occurs synchronously with compression force during normal physiological activities. Schätti et al. designed a bioreactor that utilizes a combination of shear and dynamic compression. The results showed that compression or shear alone cannot induce human BM-MSCs to differentiate into cartilage. The shear superimposed on the dynamic compression combination resulted in a significant increase in chondrogenic gene expression. Increases in sGAG and collagen were detected in only the mechanical combination group [68].

#### 3.2.3. Compressional Forces

AC, as a mechanoactive tissue, undergoes compression on a daily basis. In the processes of human movement, under the action of compression force, the relative collision of two articular surfaces in the joint causes deformation of the cartilage tissue. The magnitude, frequency, and profile of the pressure are important parameters of pressure load in bioreactor research. The average pressure of AC as the load-bearing tissue of the knee joint is approximately 0.5-7.7 MPa. Under physiological compression, the average shape variable of AC can be up to approximately 1/8 of its thickness [69, 70]. Cartilage tissue includes specific chondrocyte sequences and extracellular matrix components for physiological compression. At present, most of the research on mechanical stimulation and bioreactors is related to compression force. Studies have shown that compression is beneficial for chondrocytes to upregulate the synthesis of ECM components [71, 72], promote the proliferation of stem cells [72], and increase the compression modulus of cells/scaffolds [73]. Other studies have pointed out that mechanical stimulation can induce the migration of stem cells and chondrocytes under the condition of intermittent compression [74, 75]. Anderson et al. searched the PubMed database to identify relevant publications up to 2017 and found that there is little standardization of research work in the field of dynamic stress-induced chondrogenesis. In most of the studies searched, dynamic loading was found to have positive effects on chondrogenic gene expression, biomechanical modulus, and proteoglycan content, in contrast to its effect on collagen content [76]. Chong et al. found that 20% compression can enhance the biosynthetic activity of human chondrocytes from osteoarthritic joints, which suggests that dynamic compression is equally effective as static compression for the biosynthesis of chondrocytes from osteoarthritis [77].

For the design of compression bioreactors, the reactor provides a controlled environment for assessing the impact of cartilage compression. For compression load bioreactor design, it is necessary to determine the compression state (dynamic or static compression) as well as its strain amplitude, duration, and frequency. By changing the strain and duration, Chen et al. determined that the optimal dynamic culture conditions of chondrocytes in refrigerated gel scaffolds under a cyclic dynamic compression load of 1 Hz were as follows: 20% strain and 3 h/d stimulation time. In addition, the feasibility and effectiveness of mechanical stimulation to promote chondrogenic differentiation of ASCs through coculture of chondrocytes and ASCs in vitro were confirmed [78].

### 3.3. Noncontact Physical Stimulation

#### 3.3.1. Electromagnetic Field (EMF) Stimulation

Electromagnetic fields (EMFs) are another extrinsic factor in the culture of cells and tissues. Due to the piezoelectricity of hyaline cartilage's extracellular matrix, natural articular cartilage can convert electromagnetic oscillations into mechanical oscillations and vice versa [79]. As mentioned above, recent studies have found that EMFs can affect chondrocyte proliferation and articular chondrocyte extracellular matrix formation [29, 79]. Based on how magnetic fields change over time, EMFs can be divided into static magnetic fields (SMFs) and pulsed EMFs (PEMFs). An SMF has a constant magnetic field, while PEMFs have a certain pulse frequency. Ciombor et al. used the demineralized bone matrix- (DBM-) induced endochondral ossification model to show the effects of PEMFs stimulation. The results show that low-frequency EMF can enhance the chondrogenic differentiation in an endochondral ossification model [80]. To the contrary, Wilmot et al. exposed the cartilage layer of rat joints to PMEF with a frequency of 75 Hz. The results showed that the electromagnetic field had a negative effect on the culture of cartilage tissue [81]. In addition to frequency, the strength of the electromagnetic field is also an important parameter. Štolfa et al. found that a 0.6 T static magnetic field (SMF) increased metabolic activity in human cartilage tissue [82], whereas Hsieh et al. determined that a high-intensity SMF [3 Tesla (T)] reduced human chondrocyte cell proliferation and induced cell apoptosis [83]. In addition, many studies have shown that the moderate-intensity SMFs (1 mT to 1 T) can increase the proliferation of cells [84, 85]. For example, Jaberi et al. studied the effects of a moderate-intensity permanent magnetic field (40 mT) on cartilage repair in an animal model. The result showed that a moderate-intensity SMF (40 mT) enhanced the repair of cartilage damage in rabbits [30].

In brief, for the design of magnetic bioreactors, it is vital to determine the type, intensity, and frequency of electromagnetic fields promoting the behavior of cells in a favorable direction.

#### 3.3.2. Ultrasonic Stimulation

With the wide application of ultrasound (US) in the industrial and biomedical fields, its interaction with living cells and tissues has been extensively studied. The regulation of cells by US is bidirectional, and in vitro studies have shown that high-intensity US can induce cell death through lysis, necrosis, or apoptosis [86, 87]. Therefore, we need to consider many factors when using US, such as the prediction of cell death model and control of US intensity, to obtain the biological effects we need to the maximum extent and minimize the adverse effects. Recent studies have shown that low-intensity continuous ultrasound (LIUS) has critical effects on cartilage metabolism, cartilage repair, and the culture of chondrocytes and mesenchymal stem cells (MSCs). For example, Zhang et al. studied the effects of pulsed LIUS (1.5 MHz) on chondrocyte culture. The results showed that the LIUS increased the expression of collagen II and inhibited the development of chondrocyte hypertrophy [88].

LIUS is also being widely applied in AC tissue engineering combined with the 3D culture of chondrocytes and MSCs. For instance, Hasanova et al. used low-intensity diffuse ultrasound (5.0 MHz, 0.14 mW/cm^2^) to stimulate bovine chondrocytes seeded in three-dimensional (3D) chitosan-based scaffolds and evaluated the expression levels of chondrocyte-specific genes. In conclusion, it suggested that US stimulation can modulate the proliferation and expression of specific genes of chondrocytes seeded in 3D matrices [89]. Therefore, based on the positive bioeffects of LIDUS and their applications to the field of cartilage tissue engineering, we can design and develop an ideal ultrasonic bioreactor.

LOT: low oxygen tension; HP: hydrostatic pressure; hMSCs: human mesenchymal stromal cells; hBMSCs: human bone marrow mesenchymal stromal cells; ASCs: adipose-derived stromal cells; PRF: platelet-rich fibrin; CPP: calcium polyphosphate; rBMSCs: rat bone marrow stromal cells; CS: chitosan; SF: silk fibrin; n-HA: nano-hydroxyapatite; pBM-MSCs: porcine bone marrow-derived mesenchymal stem/stromal cells; PEMFs: pulsed electromagnetic fields; SMF: static magnetic field; LIUS: low-intensity continuous ultrasound.

## 4. Application of Existing Bioreactors in Combination with Other Emerging Technologies

### 4.1. 3D-Printed (3DP) Bioreactors

Current bioreactor systems face problems of limited customization capability, high cost, and lack of force measurement capability. It seems feasible to design and manufacture a low-cost custom bioreactor system using 3D printing technology. We can use 3D printing technology to build a culture chamber, maintain cells and engineered tissue in the medium, and place TEAC using custom grips.

Abigail et al. developed a 3DP stretch bioreactor with potential applications for multiple tissues. The group developed custom software to independently control three actuators and monitor the weighing elements for high-throughput loading experiments and to evaluate the mechanical properties of the organization under development. The results of this in vitro study and mechanical evaluation indicated that 3DP technology is a viable platform for the development of customizable, low-cost, and multifunctional mechanical bioreactor systems [90].

### 4.2. Bioreactors Using Microcarriers

One limitation of traditional flask or multilayer cell culture is its low surface to volume ratio, which often leads to an insufficient number of available cells. However, in the field of tissue-engineered cartilage regeneration, there are some seed cells, such as synovial-derived mesenchymal stem cells (SF-MSCs) [91], which are lacking in organization. Their specific chondrogenic differentiation potential is also important for cartilage regeneration [92]. Therefore, it is necessary to proliferate in the coculture system to produce experimentally and clinically usable numbers of MSCs. Van Wezel introduced the microcarrier culture system in 1967 [93]. The combination of a bioreactor with a microcarrier can improve cell proliferation while reducing time, cost, and manual operation and significantly improve relative repeatability. Microcarriers can enhance cell adhesion and suspend cells in the culture system, which greatly increases the number of cultured cells [91, 94]. As early as 1997, Baker et al. cocultured bovine chondrocytes and Cytodex-3 microcarriers in an RWV. This experiment showed that the three-dimensional environment of the coculture system played a role in the growth, differentiation, and ECM formation of bovine chondrocytes [95]. Another advantage of the microcarrier is that the cell-implanted microcarrier can be transferred directly to the site of repair, avoiding the steps of collecting cells and planting them on the scaffold [96]. Lam et al. reported that biodegradable poly (epsilon-caprolactone) microcarriers combining bioreactors greatly increased the production of hMSCs and cell secretion factors [97].

Bioreactor and microcarrier coculture systems can not only reduce costs but also promote more efficient liquid oxygen exchange and maintenance of the extracellular environment. In addition, the system can regulate the oxygen partial pressure and pH of the cell environment acting on the mechanical loading of the cell. Therefore, the system can effectively manage and regulate tissue-engineered cells.

## 5. Conclusion and Future Perspectives

Bioreactors have been used to improve the quality of cartilage tissue engineering by importing physiological environmental factors present in natural AC, such as oxygen and pH, nutrient delivery, and mechanical loading. The potential of bioreactors in tissue engineering is clear, although the role of stimulation by various mechanical loads in directing cellular behavior is controversial. In addition, various new technologies and strategies have been applied in combination with bioreactors, not only greatly improving the efficiency of the bioreactor but also ultimately enabling the construction and culture of high-quality tissue-engineered cartilage.

Despite this, further investigation is needed to elucidate the specific biochemical and biomechanical factors required for the development of cells, tissues, or organs. Results and parameters of theoretical research are indispensable for the design of bioreactors, which is beneficial to fully understand the regulatory mechanisms of cartilage growth and differentiation in order to produce successfully engineered tissues with the best characteristics. Most importantly, the basic mechanical biology should also be explored to enhance this achievement. In addition, there are also some new ideas for bioreactors, such as the proposal of the in vivo bioreactor, which provides a promising approach to provide in vivo conditions for cartilage engineering. Finally, the combination of tissue engineering and advanced technologies is not only the development trend of tissue engineering but also the development direction of bioreactors. We believe that the application of bioreactors in cartilage tissue engineering, especially the customized construction of in vitro engineered cartilage, will play a significant role in the personalized treatment and prognosis of clinical cartilage damage repair in the future.

## Figures and Tables

**Table 1:** Compilation of studies that investigated the responses of seeded cells under various bioreactors.

Authors	Cell source	Study design	Type of bioreactor	Results
Xu et al. [23]	Chondrocytes	Alginate gel beads with chondrocytes	Spinner flasks	Improved the GAG quantification and relevant gene expression
Yoon et al. [24]	hADSCs	Cellular metabolic response to dynamic loading	Spinner flasks	Enhanced chondrogenic differentiation of hADSCs
Zhu et al. [25]	hADSCs	Chitosan/gelatin hybrid hydrogel and subsequent dynamic loading	Rotating wall vessels	Enhanced proliferation and matrix secretion
Nordberg et al. [26]	RCS	Culture of cell pellets	Rotating wall vessels	Mechanical stimulation influenced the expression of LRP4/5/6 in chondrocytes
Theodoridis et al. [27]	hADSCs	3D-printed PCL scaffolds seeded cells	Perfusion bioreactors	Achieved better penetration and uniform distribution of the cells within the scaffold
Pigeot et al. [28]	hBM-MSCs	Human MSCs cultured in the perfusion bioreactor	Perfusion bioreactors	Generated a homogeneous hypertrophic cartilage ECM; efficiently induced towards apoptosis
Dikina et al. [31]	hBM-MSCs	Scaffold-free human mesenchymal stem cell sheets in response to variable magnetic fields	Magnetic bioreactors	Did not affect cartilage formation
Thakurta et al. [34]	Chondrocytes	Chondrocyte-seeded scaffolds	Ultrasonic bioreactors	Ultrasonic stimulation significantly impacted cell proliferation and depth-independent cell population density
Subramanian et al. [36]	Chondrocytes	Studying the response of cells to ultrasonic stimulation	Ultrasonic bioreactors	Positively influenced several factors, including cell proliferation, viability, and gene expression of select chondrocytic markers

**Table 2:** Summary of responses of seed cells to different design factors in bioreactors.

Authors	Cell+scaffold type	Design condition	Loading parameters	Results
Kean et al. [37]	Human chondrocytes+porcine devitalized synoviocyte matrix scaffold	LOT	5%	Promote chondrogenesis
Shearier et al. [38]	hMSCs spheroids+scaffold-free	LOT	2% 4 days	Maintain stemness Upregulate the synthesis of ECM components and growth factors
Shi et al. [39]	Rat ASCs and chondrocytes+scaffold-free	LOT	2% 3 days	Promote chondrogenic differentiation Enhance proliferation
Yasui et al. [40]	Human synovial MSCs+scaffold-free	LOT	5% 200 days	Prevent senescence Promote chondrogenic differentiation Enhance proliferation
Hwang et al. [41]	Human ADSCs+scaffold-free	LOT	1% 2 days	Increase migration Promote chondrogenic differentiation Decrease osteogenic differentiation
Cheng et al. [53]	Rabbit BMSCs+PRF membrane scaffold	HP	0.12 MPa 1 h 4 days	Promote proliferation and chondrogenic differentiation
Shahmoradi et al. [54]	hBMSCs+human demineralized bone matrix scaffold	Cyclic HP	5 MPa 0.5 Hz 4 h 7 days	Improve chondrogenic differentiation
Hodder et al. [52]	3D cultured chondrocytes+scaffold-free	Static HP	≤5-10 MPa ≥14 days	Improve generation of cartilage
Nazempour et al. [59]	Bovine chondrocytes+agarose scaffold	Oscillating HP Fluid shear	0.02 Pa, 21 days for shear 0.5 Hz, 4 MPa, 4 h/day, 15 days for OHP	Protect cartilage Enhance extracellular matrix synthesis
Theodoropoulos et al. [60]	Bovine chondrocytes+cylinders of CPP scaffold	Tissue shear	90 rpm 28 days	Alter expression of integrin
Grad et al. [61]	Bovine chondrocytes+polyurethane scaffold	Tissue shear	1 Hz 1 h 21 days	Regenerate and maintain functional joint surface Support mechanical capability
Jonnalagadda et al. [62]	Human articular chondrocytes+scaffold-free	Oscillating shear	1-50 Hz 21 days	Promote hyaline cartilage formation
DiFederico et al. [71]	Bovine chondrocytes+agarose Scaffold	Dynamic compression	15% strain 1 Hz 2 days	Upregulate the synthesis of ECM components
Guo et al. [72]	hMSCs encapsulated alginate hydrogel beads+scaffold-free	Dynamic compression	0.5 Hz 0-6813 Pa 21 days	Upregulate the synthesis of ECM components Promote proliferation
Wang et al. [73]	rBMSCs+CS/SF/n-HA scaffold	Periodic dynamic compression	10% strain 0.5 Hz 2 h action + 4 h pause/cycle, 4 cycles/day 14 days	Increase compression modulus
Gamez et al. [75]	pBM-MSCs+alginate scaffold	Periodic dynamic compression	10% strain 0.3 Hz 180 cycle (10 mins) action + 10 s pause 1 day	Induce migration
Chong et al. [77]	Human osteoarthritic chondrocytes+agarose scaffold	Uniaxial dynamic compression	20% strain 1 Hz Sinusoidal waveform 8 days	Enhance biosynthetic activity of osteoarthritic chondrocytes
Ciombor et al. [80]	Chondrocytes+scaffold-free	PEMFs	Low-frequency EMF	Enhance the chondrogenic differentiation
Wilmot et al. [81]	The cartilage layer of rat joints+scaffold-free	PEMFs	75 Hz PEMF	Negative effect on the culture of cartilage tissue
Štolfa et al. [82]	Human cartilage tissue+scaffold-free	SMF	0.6 T SMF	Increase metabolic activity
Hsieh et al. [83]	Human chondrocyte+scaffold-free	SMF	3 T SMF	Reduce cell proliferation and induced cell apoptosis
Zhang et al. [88]	Chondrocytes+scaffold-free	LIUS	1.5 MHz	Increase the expression of collagen II and inhibit the development of chondrocyte hypertrophy
Hasanova et al. [89]	Bovine chondrocytes+chitosan Scaffold	LIUS	5.0 MHz	Modulate the proliferation and expression of specific genes
